# Ozone in Botulinum Toxin–Induced Ptosis: Some Comments

**DOI:** 10.1111/jocd.70850

**Published:** 2026-04-07

**Authors:** Dario Bertossi, Marianno Franzini, Luigi Valdenassi, Umberto Tirelli, Salvatore Chirumbolo

**Affiliations:** ^1^ Department of Surgery, Dentistry, Paediatrics and Gynaecology, Unit of Maxillo‐Facial Surgery University of Verona Verona Italy; ^2^ Italian Scientific Society of Oxygen‐Ozone Therapy (SIOOT) and High Master School of Oxygen‐Ozone Therapy, University of Pavia Pavia Italy; ^3^ Tirelli Medical Group, Pordenone and Former Head of the Oncology Unit Cancer National Institute Aviano (PN) Italy; ^4^ Department of Engineering for Innovation Medicine University of Verona Verona Italy

To the Editor,

The clinical commentary by Gençtürk and colleagues offers an intriguing and provocative contribution to the discussion on managing botulinum toxin–induced eyelid ptosis, a complication that, while uncommon, is distressing for patients and challenging for clinicians [1]. By describing two cases in which intradermal and intramuscular ozone therapy appeared to accelerate recovery, the authors place themselves at the intersection of aesthetic dermatology, regenerative medicine, and complementary therapeutic approaches. The report is clearly written, visually supported by clinical photographs, and framed with appropriate caution in its conclusions. At the same time, the manuscript raises several conceptual, methodological, and interpretative issues that deserve careful scrutiny before the proposed approach can be meaningfully integrated into clinical practice.

One of the strengths of the article lies in its clinical plausibility. Botulinum toxin–induced ptosis is typically self‐limiting, but its duration can be functionally and psychologically burdensome. The authors correctly outline the conventional pathophysiology, namely unintended diffusion of toxin to the levator palpebrae superioris, and they acknowledge established management strategies such as sympathomimetic eye drops [1].

Against this backdrop, the reported rapid improvement within 2–5 days is striking and, on the surface, difficult to reconcile with the expected natural course. The authors' attempt to explain this discrepancy through anti‐inflammatory and anti‐edematous effects of ozone, rather than direct toxin inactivation, is biologically coherent and avoids overstatement. Their discussion is further strengthened by references to experimental and clinical literature suggesting that low‐dose ozone exposure can modulate cytokine profiles, reduce TNF‐α, and promote tissue repair [2, 3, 4].

However, the central weakness of the report lies in its evidentiary fragility. Two uncontrolled case reports, by definition, cannot establish causality, and in this specific context the absence of any comparator is particularly problematic. Botulinum toxin–induced ptosis is known to show variability in onset, severity, and recovery time. While the authors emphasize that recovery within a few days is atypical, the literature does document occasional rapid improvement, especially in mild cases or in patients who subconsciously recruit compensatory mechanisms such as frontalis muscle activation. Without a contemporaneous control group, or even a detailed historical comparison within the same practice, it remains impossible to disentangle the true treatment effect from spontaneous resolution or regression to the mean.

The assessment of outcomes represents another important limitation. Improvement was evaluated primarily through clinical inspection and photographic documentation, supplemented by patient‐reported satisfaction. While this approach is understandable in a case report, it leaves substantial room for subjectivity. Eyelid position is highly sensitive to gaze direction, lighting, facial expression, and camera angle. The authors themselves acknowledge that objective measurements such as Margin Reflex Distance 1 were not used, but the implications of this omission are significant. In a condition where millimeters matter, even small variations can create the impression of dramatic change. The lack of blinded assessment further amplifies the risk of observer bias, especially when both clinicians and patients are aware of the intervention and motivated to perceive improvement.

The therapeutic protocol itself, although described with reasonable technical detail, raises questions about reproducibility and standardization. The choice of ozone concentration, total volume, injection sites, and session spacing appears empirically derived rather than evidence‐based. While the authors emphasize the precision of the ozone generator and its regulatory certifications, this does not substitute for a rationale explaining why 3 gamma ozone, 2.5 mL total volume, and two sessions were selected. It is unclear whether different dosing regimens would yield similar, superior, or inferior outcomes, or whether the observed effect depends critically on injection into specific muscles such as the corrugator. Without dose–response data or mechanistic validation, the protocol risks being perceived as artisanal rather than systematically developed [1].

Safety considerations, though briefly addressed, warrant deeper discussion. Ozone is indeed used in various medical contexts, but it is also a potent oxidizing agent with well‐documented toxic effects when improperly administered or inhaled. The periorbital region is anatomically delicate, rich in vasculature, and in close proximity to the globe. While no adverse events were observed in these two patients, the sample size is far too small to support reassuring conclusions about safety. Rare but serious complications would not be expected to emerge in such a limited series, and the absence of harm should therefore be interpreted with caution rather than confidence.

From a conceptual standpoint, the manuscript also sits within a broader controversy surrounding ozone therapy itself. Despite its long history and growing popularity in some regions, ozone therapy remains variably accepted across medical communities, often due to inconsistent regulatory frameworks and a paucity of high‐quality randomized trials. By positioning ozone therapy as a “promising adjunctive option,” the authors take a measured stance, yet readers may still question whether the biological plausibility presented is sufficient to overcome the general skepticism surrounding this modality. The reliance on indirect evidence from musculoskeletal conditions, such as piriformis syndrome, further underscores the gap between mechanistic speculation and condition‐specific proof.

In conclusion, the article succeeds as a hypothesis‐generating report that invites further investigation into a novel and potentially useful approach for a frustrating clinical complication. Its principal contribution lies not in establishing efficacy, but in stimulating thoughtful scientific dialogue and encouraging the transition from anecdotal observation to structured clinical investigation. Advancing this line of inquiry will require well‐designed controlled studies, objective outcome measures, standardized treatment protocols, and rigorous safety monitoring. Such methodological rigor should be viewed not as a barrier to innovation, but as the necessary pathway through which promising ideas can be responsibly validated and integrated into evidence‐based practice. Until such data are available, ozone therapy for botulinum toxin–induced ptosis should be considered investigational, an intriguing possibility that warrants careful, systematic study and prudent clinical application.

## Conflicts of Interest

The authors declare no conflicts of interest.

## Data Availability

Data sharing not applicable to this article as no datasets were generated or analysed during the current study.
